# Food insecurity and risky sexual behaviors among college students during the COVID-19 pandemic

**DOI:** 10.1186/s12889-023-16330-2

**Published:** 2023-08-15

**Authors:** Bertille Assoumou, Jennifer Pharr, Courtney Coughenour

**Affiliations:** School of Public Health, University of Nevada, 4700 S. Maryland Parkway Suite # 335, Las Vegas, NV 89119 USA

**Keywords:** Food insecurity, Risky sexual behaviors, College students, Condom use, Number of sexual partners

## Abstract

**Background:**

Sexually transmitted infections (STIs) and food insecurity are public health concerns in the United States (US) due to their growing prevalence and incidence among young people, and particularly in college students. Studies have reported that college students are at higher risk of STIs due to the high rates of risky sexual behavior (RSB). Most studies report a food insecurity prevalence of more than 30% among college students, which was more than twice the overall national food insecurity rate of 10.5% in 2020. This study aims to assess the relationship between food insecurity and RSB among college students during the early-stages of the COVID-19 pandemic.

**Methods:**

This was a cross-sectional study from a convenience sample of 320 students enrolled at the University of Nevada, Las Vegas during the 2020 Fall semester. Data was collected using an online survey. Univariate and multivariate logistic regression analyses were conducted.

**Results:**

Food insecure students were 2.9 times more likely to report receiving or giving fellatio without using a condom at least once in the past 6 months (*P* < 0.01) compared to food secure students. There was no significant association between food insecurity and other RSBs evaluated in this study.

**Conclusions:**

The current study provides valuable information on food insecurity and RSB among college students during the early stages of the COVID-19 pandemic. Larger and longitudinal studies are needed to assess the trajectory of the association between food insecurity and fellatio with no condom use and other RSB among college students.

## Background

Sexually transmitted infections (STIs) and food insecurity are public health concerns in the United States (US) due to their growing prevalence and incidence among young people in general and particularly in college students [1, 2]. The national prevalence of food insecurity among college students is unknown, but most studies report prevalence of more than 30% [2], which was more than twice the overall national food insecurity of 10.5% in 2019. Studies examining food insecurity rates during the early stages of the COVID-19 pandemic report varying results. One study conducted in May 2020 among Texas college students reported that almost 35% of respondents were food insecure [3, 4]. Estimating food insecurity from food insufficiency rates among a national sample of US adults, Schanzenbach and Pitts reported that rates doubled when comparing February 2020 estimated rates with April–May 2020 data [5]. Similarly, a national sample of low-income US adults reported that 36% were food insecure in March of 2020 [3]. However, the US Department of Agriculture (USDA) reported that overall rates of food insecurity remained stable at 10.5% throughout 2020 as a whole [6] and 10.2% in 2021 [7]. Food insecurity among college students is of concern because of the short- and long-term consequences this can have on their lives and particularly on their academic success [2]. Food insecurity among college students has been linked to depression, anxiety, and a decrease in academic performance [2, 8]. Payne et al. point out that the Risk Sensitivity Theory, a theory about animal behavior, also applies to human behavior. Like foraging animals, as humans’ perceptions of need increase, so too does their risk-taking behavior [9]. Thus, it is not unreasonable to hypothesize an association between food insecurity and RSB. Given the associations of both FI and RSB with negative physical and mental health outcomes, it is important to study the association between RSB and FI among college students.

While the national prevalence of STIs among college students is unknown, over 60% of students are between the ages of 15 and 24 years, an age group that is overburdened with STIs [1]. Half of the new STI diagnoses in 2018 were made among individuals between 15 and 24 years of age [10] and a quarter of all new Human Immunodeficiency Virus (HIV) infections were among people 13 to 24 years old [11]. Studies have reported that college students are at higher risk of STIs due to the high rates of RSBs among this population [1, 12, 13]. RSBs include having multiple sexual partners, inconsistent condom and contraceptive use, casual sexual encounters, alcohol, and drug use, among others [1]. An American College Health Association study of 94,806 students on 117 campuses, including 113 four-year institutions, found that 24.2% of college students reported having two or more sexual partners in the past academic year, 47.9% of sexually active students did not use condoms during their last vaginal intercourse, 72.3% reported not using condoms for their last anal intercourse, and 13.9% reported having unprotected sex after drinking alcohol in the past academic year [14]. Limited studies evaluated the impact of the COVID-19 pandemic on sexual behavior among college students and young people. However, findings on sexual behavior among US adults during the pandemic are mixed, with some studies reporting a decrease in hookups, casual sex, and number of sexual partners [15], and other studies reporting an increase in sexual partners [16, 17] and an increase in unprotected sex [16, 18].

Several studies have reported an association between food insecurity and some RSBs among specific populations, including people infected with HIV [19–21] and people experiencing homelessness [22]. In their study evaluating food insecurity and RSBs among homeless HIV-infected people in San Francisco, Vogenthaler and colleagues reported that there were 1.5 times higher odds of having multiple sexual partners for each five-point increase in the severity of food insecurity (as measured by the Household Food Insecurity Access Scale). Moreover, for each 5-point increase in the severity of food insecurity, participants had 2 times the odds of engaging in unprotected sexual intercourse [22]. Food insecurity was reported as a significant predictor of condom use such that higher degrees of food insecurity were associated with lower likelihood of using condoms [21]. Additionally, Loosier and colleagues reported that the following five risk indicators were associated with higher rates of food insecurity: (1) sex with male injection-drug users, (2) sex with HIV-positive men, (3) past chlamydia or gonorrhea diagnosis, (4) sex with non-monogamous partners, and (5) exchanging sex for money or drugs [23].

Although several studies have reported an association between food insecurity and some RSBs among specific populations, little is known about the relationship between food insecurity and RSBs among college students. Given the high rates of food insecurity and the high rates of STIs and RSBs among young people, it is crucial to explore the potential link between these two public health problems, especially during the COVID-19 pandemic which saw increased rates of food insecurity at the outset [3–5].

## Methods

### Methodology and data collection

The methodology and the data collection used in the present study are similar to those used in our previous study [24]. This was a cross-sectional study that collected data from a convenience sample of students from the University of Nevada, Las Vegas (UNLV) who were enrolled during the 2020 Fall semester. The online survey was hosted through the web-based survey tool Qualtrics and was available only in English. The survey was sent via a weekly email newsletter system to all UNLV students [25]. The weekly email newsletter was sent to all the students enrolled at UNLV in the Fall 2020, approximately about 31,140 [26]. Participants were limited to students 18 years of age and older. This study was deemed exempt by the UNLV Institutional Review Board.

#### Dependent variables

Sexual behaviors were the dependent variables. These were the number of sexual partners, rates of condom use, and rates of alcohol/cannabis use. Data on sexual behavior were collected using questions from the Centers for Disease Control and Prevention’s (CDC) 2018 Behavioral Risk Factor Surveillance System (BRFSS), the 2018 General Social Survey (GSS), and the 2015–2017 National Survey of Family Growth (NSFG) questionnaire. These are national surveys that use validated questionnaires [27–29]. Questions from the Sexual Risk Survey (SRS) developed by Turchik and Garske were also used. The SRS is a valid survey to measure sexual-risk-taking behaviors among college students [30].

#### Independent variable

Food security was the independent variable. Food security was measured using the six-item short form of the USDA household food security survey. This survey has strong reliability and validity, and was reported to provide results comparable to national food security statistics [31]. A Cronbach’s alpha of 0.87 has previously been reported for this survey [32]. Survey participants were classified by food security status: food secure participants were those with raw score of 0 or 1, and food insecure participants were those with a raw score between 2 and 6.

#### Covariates

Covariates included factors that have been reported in the literature as (1) risk factors of food insecurity [2] and (2) influence the relationship between food insecurity and RSBs [22, 32]. They included age, race/ethnicity, marital status, gender, sexual orientation, year in college, grade point average, employment status, time in foster care, income, household income, living with and responsible for children younger than 18 in the household, participants’ current residence (e.g., living with their parents, living independently), and participating in government assistance programs including SNAP (Supplemental Nutrition Assistance Program) and Medicaid. Most of these variables were measured using questions from the 2018 CDC Behavioral Risk Factor Surveillance System (BRFSS) and the 2018 General Social Survey (GSS).

### Analytical methods and data analysis

A total of 353 surveys were exported from Qualtrics into SPSS version 25. Of these, 33 surveys were removed because they had missing data for the dependent or independent variables.

Descriptive statistical analysis was conducted to characterize the overall study population. Chi-square tests were used for categorical variables, independent t-tests were used for continuous variables, univariate analyses were conducted, and then multivariate logistic regression analyses were conducted to test the association of covariates on both food security status and RSB. Covariates that were significantly associated with food insecurity or RSB in the univariate analysis were included in the multivariate analysis. Adjusted odds ratios (AOR) and corresponding 95% confidence intervals (CI) were computed. Statistical significance was set at P-values lower than 0.05.

## Results

### Descriptive statistics

A total of 320 students completed the survey including the dependent variable, independent variables, and covariates. Of these, 75.3% were female, 55.3% were aged 18–24 years, and 33.4% were white. Moreover, 71.9% of participants were single, 76.6% were heterosexual, 82.2% were full-time students, 58.1% worked full or part-time, 78.8% had an annual income lower than $25,000, and 54.7% lived at their parents or guardian’s home. The mean GPA was 3.48 with a standard deviation of 0.573 (see Table 1 for all demographic information).

**Table 1:** Summarized demographic characteristics of a subsample of University of Nevada, Las Vegas Students, Fall 2020

	**Characteristics**	**Frequency**	**Percentage (%)**
**Age**	18–24 years	177	55.3
	25 or more	90	28.1
	Missing	53	16.6
**Race/Ethnicity**	White	107	33.4
	Other	213	66.6
**Marital Status**	Single	230	71.9
	Other	89	27.8
	Missing	1	0.3
**Gender**	Female	241	75.3
	Other	78	24.4
	Missing	1	0.3
**Sexual Orientation**	Heterosexual or Straight	245	76.6
	Other	74	23.1
	Missing	1	0.3
**Year in College**	Year 1–3 undergraduate	193	60.3
	Over year 3 undergraduate and others	126	39.4
	Missing	1	0.3
**Enrollment Status**	Full time	263	82.2
	Part time or Other	57	17.8
**First Generation College Student**	Yes	159	49.7
	No	161	50.3
**Employment Status**	Unemployed	134	41.9
	Working full or part time	186	58.1
**Annual Income**	< $25,000	252	78.8
	> $25,000	66	20.6
	Missing	2	0.6
**Household Income**	< $50,000	144	45
	> $ 50,000	173	54.1
	Missing	3	0.9
**Government Assistance Programs**	Yes	54	16.9
	None	261	81.6
	Missing	5	1.6
**Smoking**	Yes	19	5.6
	No	298	93.1
	Missing	3	0.9
**General Health**	Excellent or Very Good	141	44.2
	Other	178	55.6
	Missing	1	0.3
**Living with and Responsible for children under 18 years**	Yes	40	12.5
	No	279	87.2
	Missing	1	0.3
**Time in Foster Care**	Yes	7	2.2
	No	311	97.2
	Missing	2	0.6
**Disability**	Yes	39	12.2
	No	280	87.5
	Missing	1	0.3
**Residence**	Parent/guardian's home	175	54.7
	Other	144	45
	Missing	1	0.3

#### Prevalence and characteristics of food insecurity and risky sexual behaviors

A total of 29.4% of the study participants were food insecure. Overall, 65% of the participants were sexually active in the past 12 months; however, only 19.9% had multiple sexual partners (2 or more). A total of 71.8% of participants did not use a condom at their last sexual intercourse, 74.9% reported engaging in vaginal intercourse without the use of a condom at least once in the past six months, 73.4% of participants reported giving or receiving fellatio without using a condom at least once in the past 6 months, and 17.5% of students reported engaging in anal sex without using a condom at least once in the past six months. Overall, 51.5% of the sexually active participants reported using alcohol before or during sex in the past six months, and 29.3% of the sexually active participants reported using cannabis before or during sex in the past six months (see Table 2).

**Table 2:** Prevalence of food insecurity and risky sexual behaviors of a subsample of University of Nevada, Las Vegas Students, Fall 2020

	**Characteristics**	**Frequency**	**Percentage (%)**
**Food Security Status**	Food Secure	219	70.6
	Food Insecure	91	29.4
**Sexual Activity Past 12 Months**	Not Sexually Active	73	22.8
	Sexually Active	209	65.3
**Number of Sexual Partners in the Past 12 Months**	0 or 1 partner	226	80.1
	Multiple	56	19.9
**Condom Use at last Sexual Intercourse**	Yes	59	28.2
	No	150	71.8
**Condom use in the past 12 months**	Every time/Most of the times	55	26.3
	Half the time, Sometimes, None of the times	154	73.7
**Vaginal Intercourse without Condom in the Past 6 Months**	0 times	52	25.1
	1 or more times	155	74.9
**Fellatio without Condom in the Past 6 Months**	0 times	55	26.6
	1 or more times	152	73.4
**Anal Sex without Condom in the Past 6 Months**	0 times	170	82.5
	1 or more times	36	17.5
**Alcohol use before or during sex in the Past 6 Months**	0 times	100	48.5
	1 or more times	106	51.5
**Cannabis use before or during sex in the Past 6 Months**	0 times	145	70.7
	1 or more times	60	29.3

#### Risky sexual behaviors by demographic characteristics

After conducting a Chi-square test for categorical variables and independent samples t-tests for continuous variables, there was a significant association between sexual activity in the past 12 months and age (*p* = 0.02), marital status (*p* < 0.01), year in college (*p* = 0.04), employment status (*p* = 0.01), annual income (*p* = 0.01), smoking status (*p *= 0.02), general health (*p* = 0.03), residence (*p* < 0.01), and GPA (*p* < 0.01). The number of sexual partners was significantly associated with marital status (*p* < 0.01) and GPA (*p* < 0.01).

There was a significant association between condom use at last sexual intercourse and age (*p* = 0.04), marital status (*p* < 0.01), and gender (*p* = 0.01). Moreover, there was a significant association between condom use in the last 12 months and age (*p *= 0.03), marital status (*p* < 0.01), gender (*p* < 0.01), and year in college (*p* < 0.01). Vaginal intercourse without a condom in the past six months was significantly associated with marital status (*p* = 0.02), gender (*p* < 0.01), sexual orientation (*p* < 0.01), year in college (*p* = 0.04), and GPA (*p* = 0.04). Giving or receiving fellatio without condom in the past six months was significantly associated with general health (*p* = 0.02), and anal sex without condom in the past six months was significantly associated with race/ethnicity (*p *= 0.04), sexual orientation (*p* = 0.02), and smoking (*p* = 0.02).

Alcohol use before or during sex in the past six months was significantly associated with year in college (*p* = 0.01), annual income (*p* < 0.01), general health (*p* = 0.02.), living with and responsible for children under 18 years (*p* = 0.02), and residence (*p* = 0.03). Additionally, there was a significant association between cannabis use before or during sex in the past six months and age (*p* < 0.01), year in college (*p* = 0.01), annual income (*p* = 0.04), smoking (*p* < 0.01), and residence (*p* < 0.01).

### Univariate logistic regression analysis of food security status and risky sexual behaviors

Table 3 shows the results of the univariate logistic regression analyses. There was no significant relationship between food insecurity and most RSBs, but it was associated with giving or receiving fellatio without a condom in the past six months. Food insecure students were 2.9 times more likely to report that they received or gave fellatio with no use of a condom at least once in the past 6 months (*p* < 0.01) compared to food secure students.

**Table 3:** Univariate logistic regression analysis of food security status and risky sexual behaviors

	**Sexually Active in the Past 12 Months** **OR (95% CI), *P*-Value**	**Multiple Sexual Partners in the last 12 months** **OR (95% CI)**	**No Condom Use at last Sexual Intercourse** **OR (95% CI), *P*-Value**	**Condom use in the last 12 months: Half the time, sometimes, none of the times** **OR (95% CI), *P*-Value**	**Vaginal Intercourse with no condom in the past 6 Months** **OR (95% CI), *P*-Value**	**Given or Received Fellatio without Condom in the Past 6 Months** **OR (95% CI), *P*-Value**	**Anal Sex without Condom in the Past 6 Months** **OR (95% CI), *P*-Value**	**Alcohol Use Before or During Sex in the past 6 Months** **OR (95% CI), *P*-Value**	**Cannabis Use Before or During Sex in the past 6 Months** **OR (95% CI), *P*-Value**
**Food Security Status**									
Food Secure	Reference	Reference	Reference	Reference	Reference	Reference	Reference	Reference	Reference
Food Insecure	1.49 (0.80–2.75), 0.21	1.33 (0.71–2.48), 0.37	1.47 (0.74–2.90), 0.27	1.65 (0.81–3.33), 0.17	1.47 (0.72–2.99), 0.29	2.90 (1.32–6.37), < 0.01	1.53 (0.72–3.22), 0.27	1.05 (0.58–1.9), 0.86	0.78 (0.4–1.53), 0.47

### Multivariate logistic regression analysis of food security status and fellatio without condom use in the past six months

Table 4 shows the results of the multivariate logistic regression analysis of giving or receiving fellatio without condom use in the past six months, food security status, and demographic variables. After including the covariates, food insecure students were 2.89 times more likely to report giving or receiving fellatio without condom use in the past six months (*p* = 0.03) than food secure students.

**Table 4:** Multivariate logistic regression assessing the relationship between having given or received fellatio without condom in the past 6 months and food security status, adjusting for demographic variable**s** from a subsample of University of Nevada, Las Vegas Students, Fall 2020

	**Given or Received Fellatio without Condom in the Past 6 Months** **AOR (95% CI), *P*-Value**
**Food Security Status**	
Food Secure	Reference
Food Insecure	2.89 (1.09–7.6), 0.03
**Sexual Orientation**	
Heterosexual or Straight	Reference
Other	0.49 (0.22–1.11), 0.09
**First Generation College Student**	
Yes	Reference
No	0.83 (0.41–1.65), 0.59
**Household Income**	
< $ 50,000	Reference
> $50,000	1.02 (0.49–2.16), 0.95
**Government programs**	
Yes	Reference
None	0.78 (0.26–2.31), 0.65
**General Health**	
Excellent or Very Good	Reference
Other	1.73 (0.84–3.56), 0.13
**Disability**	
Yes	Reference
No	0.63 (0.18–2.12), 0.45
**Residence**	
Parent/guardian's home	Reference
Other	0.68 (0.33–1.4), 0.29

## Discussion

Few studies have examined the relationship between food insecurity and RSBs. To our knowledge, no published study has examined this relationship among college students, despite the higher rates of these two public health problems among this population. The current study uncovered that food insecure students were 2.89 times more likely to report giving or receiving fellatio without condom use in the past six months than food secure students, even after including covariates. Although there are no studies that evaluated food insecurity and condom use during fellatio among college students, a few studies have assessed the relationship between food insecurity and condom use among other populations. The results of the current study are similar to those that have examined other populations. For example, food insecurity was associated with higher odds of engaging in unprotected sex among marginally housed and homeless HIV-infected individuals living in San Francisco [22]. Food insecurity was also associated with a lower likelihood of condom use among HIV-infected people [21, 33].

The association between food insecurity and history of fellatio without condom use may be linked to the overall low rates of condom use during oral sex among adolescents and young adults in the US, as reported in other studies [34–36]. For example, one study examining the prevalence and correlates of condom use during oral sex during heterosexual intercourse from a nationally representative sample of US adults aged 15 to 44 found that only 7% of men and 6% of women reported using condoms during last oral sex [34]. Relatedly, using the same nationally representative sample but restricting the age to 15 to 24 years olds, 7.6% of females and 9.3% of males reported using a condom during last heterosexual oral sex [35]. Using the 2007 National College Health Assessment data, Buhi and colleagues reported that black college students were significantly more likely to report using a condom during last oral sex (10%) compared to white students (3.5%) [36]. While rates of condom use during oral sex are historically low, the association with food insecurity is relevant. One potential explanation for the association between food insecurity and history of fellatio without condom use among college students in the current study may be that food insecure students are more anxious about finding food and meeting their basic needs than they are about practicing safe sex. Further studies are needed to fully understand this phenomenon.

The results of the current study are different from other studies that did not find a significant association between food insecurity and condom use among recently-released prisoners [37] and among adolescents in Ghana [38]. However, these studies looked at condom use in general rather than condom use during fellatio. These differences in findings may be explained by the difference in population, the specific sample, study design, and geographic areas, but further research is needed to fully understand these differences.

There was no significant relationship between food insecurity and other RSBs evaluated in this study. These results are different compared to studies that found a significant relationship between food insecurity and multiple sexual partners [22, 39] and alcohol and drug use [21, 39, 40]. The difference in findings may, again, be due to the low sample size of the current study, the study design, or some characteristics specific to this subset of students.

This study was conducted in Fall 2020, still relatively early in the COVID-19 pandemic. Because some studies found elevated rates of food insecurity in this time frame, we hypothesized that this inability to meet basic needs may result in increases in RSBs, as life stress has been associated with increased RSBs [40, 41]. While we did not find significant associations for many RSBs and food insecurity, we are lacking a baseline from college students in our sample to detect any changes in these associations prior to and during the pandemic. Additionally, college students may have had unique circumstances and/or experiences during this phase of the pandemic that may have increased their ability to meet their basic needs. Over half of our sample lived at home with their families, which may have buffered some of the impact experienced by other populations related to food insecurity. Additionally, previous findings have reported that significant percentages of college students moved back home during the pandemic [42]. Other factors that may have influenced basic needs include the supplemental income, stimulus checks, and increased unemployment income that most US residents began receiving a few months into the pandemic.

Furthermore, the pandemic may have altered regular sexual behaviors. In general, the pandemic was associated with greater frequency of sexual intercourse [41, 43], and lower use of contraception [41]. Additionally, the COVID-19 pandemic was also associated with a decrease in opportunities to have sexual intercourse [42]. Among college students, the response to the COVID-19 pandemic and the confinement led to most students moving in with their parents and most students practicing social distancing, reducing outings including clubs and parties. These may have influenced not only student’s sexual behavior and the relationship between food insecurity and RSB. It is challenging to evaluate the effect that the COVID-19 pandemic may have had on this population and on the overall study. The results of the current study highlight the need for more studies to evaluate the relationship between food security and RSBs among college students.

This study does provide valuable information on the prevalence and characteristics of food insecurity and RSBs, and the relationship between these two public health problems among this sample of students from a university in the Southwest. However, this study is subject to a few limitations. The first limitation of this study is a response rate of less than 2% from the total university students and an overall sample size of about 320 students. There may be differences in the characteristics of the students who participated in this study compared to the overall student body. Further, participants were all respondents from one university in the Southwest US. This limits the generalizability of the current study. The cross-sectional study design of the current study does not allow for assessment of causality. Additionally, social desirability bias may have been present given the sensitive nature of sexual behavior. Potential recall bias and self-selection bias are additional limitations to this study.The current COVID-19 pandemic may have impacted the response rate, the students’ sexual behavior, and food insecurity rates. The COVID-19 pandemic and the subsequent lockdown and social distancing mandates may have at least in part influenced the student’s willingness to participate.

## Conclusion

This study provides valuable information about food insecurity and RSB among college students. Future studies should continue to assess trends in RSBs and food insecurity as operations and behaviors begin to resemble pre-pandemic levels once again. Additionally, considering the high prevalence of food insecurity and RSBs among this sample of college students, more interventions aimed at raising awareness about food pantries and other existing programs that provide free food or low-cost food to college students are needed as well as sexual education. Further, more upstream interventions are urgently needed. Universities may implement or bolster existing efforts to enroll eligible students in the SNAP and other government assistance programs to help alleviate food insecurity. Relatedly, changes to the SNAP exemptions that are specific to college students were temporarily waived during the pandemic, making more students eligible. Examination into the impact this had on college student food insecurity is warranted and permanent changes to such eligibility exemptions should be reexamined. Universities may also pilot interventions examining emerging and innovative upstream intervention efforts, such as improvements to financial literacy [44].

## Data Availability

The datasets used and analyzed during the current study are available from the corresponding author on reasonable request.

## Abbreviations

CDC: Centers for Disease Control and Prevention
HIV: Human Immunodeficiency Virus
RSB: Risky Sexual Behaviors
SNAP: Supplemental Nutrition Assistance Program
STI: Sexually Transmitted Infection
UNLV: University of Nevada, Las Vegas
US: United States
USDA: United States Department of Agriculture
